# The impact of income inequality and national wealth on child and adolescent mortality in low and middle-income countries

**DOI:** 10.1186/s12889-017-4310-z

**Published:** 2017-05-11

**Authors:** Joseph L. Ward, Russell M. Viner

**Affiliations:** UCL Great Ormond Street Institute of Child Health, 30 Guilford Street, London, WC1N 1EH UK

**Keywords:** Income inequality, Child and adolescent health, Low and middle-income countries

## Abstract

**Background:**

Income inequality and national wealth are strong determinants for health, but few studies have systematically investigated their influence on mortality across the early life-course, particularly outside the high-income world.

**Methods:**

We performed cross-sectional regression analyses of the relationship between income inequality (national Gini coefficient) and national wealth (Gross Domestic Product (GDP) averaged over previous decade), and all-cause and grouped cause national mortality rate amongst infants, 1–4, 5–9, 10–14, 15–19 and 20–24 year olds in low and middle-income countries (LMIC) in 2012. Gini models were adjusted for GDP.

**Results:**

Data were available for 103 (79%) countries. Gini was positively associated with increased all-cause and communicable disease mortality in both sexes across all age groups, after adjusting for national wealth. Gini was only positively associated with increased injury mortality amongst infants and 20–24 year olds, and increased non-communicable disease mortality amongst 20–24 year old females. The strength of these associations tended to increase during adolescence.

Increasing GDP was negatively associated with all-cause, communicable and non-communicable disease mortality in males and females across all age groups. GDP was also associated with decreased injury mortality in all age groups except 15–19 year old females, and 15–24 year old males. GDP became a weaker predictor of mortality during adolescence.

**Conclusion:**

Policies to reduce income inequality, rather than prioritising economic growth at all costs, may be needed to improve adolescent mortality in low and middle-income countries, a key development priority.

**Electronic supplementary material:**

The online version of this article (doi:10.1186/s12889-017-4310-z) contains supplementary material, which is available to authorized users.

## Background

The wealth of a society and the distribution of wealth within that society both strongly influence population health [1]. National wealth heavily influences life expectancy, and child and adolescent mortality [2]. A recent meta-analysis of national income and child mortality amongst developing countries found that a 10% increase in GDP was associated with a 10% decrease in infant mortality [3]. There is some evidence that the strength of this relationship varies by age and is weaker during adolescence than earlier childhood [4].

Societies with greater differences in income distribution have multiple worse health outcomes that include reduced life expectancy [5], higher levels of violent crime and murder [6] increased levels of obesity [7], increased infant mortality [8] and poor self reported health, after adjusting for societal wealth and poverty levels. This association has been demonstrated in both high income and developing countries [9–13] and in the latter, income inequality has also been implicated in increasing malnutrition and stunting prevalence in children under 5 [13, 14]. Although it has been suggested this observation reflects a “statistical artefact” due to the concave association between income and health [15], this has been challenged [16], and there is increasing evidence that the relationship is causal [1].

Although the impact of income inequality on health appears to differ throughout the life course, there is little agreement as to which age groups are affected most. One study found the effect of inequality on mortality to be greatest between 15 and 65 [4], whereas others have shown it to reduce after infancy [17], or after 25 amongst males [18]. It is possible that changing patterns of causes of mortality may be responsible for changes in the relationship of inequality with mortality with age, yet no previous studies have investigated this.

We undertook a systematic study of the associations of national income inequality and wealth with all-cause and high-level cause mortality groups stratified by age across the early life-course. We confined our analyses to low and middle-income countries (LMIC) as the impact of national wealth [19] and income inequality [4] on mortality are known to vary by level of economic development, yet the majority of previous studies exploring these associations have used data from high-income settings. Further, many LMIC have experienced rapid economic growth in recent years, which can be beneficial for child and adolescent health, but may also exacerbate inequalities. LMIC also have some of the highest levels of income inequality globally, and the United Nations Development Programme (UNDP) estimate this has increased by 11% between 1990 and 2010 [20]. This may have worsened child and adolescent health directly or restricted the potential benefits of economic development in these countries.

Ours is the first systematic investigation of the effect of income inequality and national wealth on child and adolescent mortality in LMIC. Previous studies of this association within developing countries have been limited by the paucity of reliable long series mortality data in LMIC. The availability of new data sources, including age and sex specific mortality estimates for 188 countries provided by the Institute of Metrics and Health Evaluation (IHME) [21] and improved estimates of income inequality data within LMIC [22], have provided the opportunity for this study.

## Methods

We examined whether income inequality and national wealth were associated with mortality amongst children, adolescents and young adults (0 to 24 years) in LMIC in 2012, the most recent year with available income inequality data.

### Mortality data

We used age and sex specific mortality data published by the Institute of Health Metrics and Evaluation (IHME), which provide mortality estimates per 100,000 population for 188 countries in 5 year age groups. Here we used data on infants, 1–4, 5–9, 10–14, 15–19 and 20–24 year olds by sex. IHME mortality estimates use data systematically identified for each country from multiple sources including surveys, censuses and vital registration systems, sibling history survey data, sample registration data, and household recall of deaths. These sources are then synthesized using spatiotemporal regression and Gaussian process regression using income per person, years of education, HIV/AIDS mortality, and country random effects as covariates [23, 24]. These data are available from www.healthdata.org/gbd/data and were accessed on 10th Feb 2016. We classified cause specific mortality using the Global Burden of Disease (GBD) categories into high-level groups i.e. communicable, maternal, neonatal and nutritional diseases (group 1, referred to here as “communicable disease”); non - communicable disease (group 2, NCD) and injuries (group 3) [25]. Mortality estimates were log transformed for regression models.

### Measure of income inequality

To determine country level income distribution we used Gini coefficients, the most commonly used summary statistic of income inequality. Gini coefficients are derived from the Lorenz curve framework, and show the percentage of income earned by the cumulative percentage of the population. In a completely equal society, the Gini coefficient would be zero and income earned within a section of the population would be proportional to the size of that population. As income distribution becomes less equal, the Lorenz curve deviates from the line of equality, up to a Gini coefficient of 1 (perfect inequality), where one person within a society receives all the income [26]. We retrieved Gini coefficients from the “all the Ginis” dataset, an international compilation of nine separate sources of Gini coefficients covering 1950 to 2012, all obtained from nationally representative household surveys [22]. Estimates were standardized for comparability and represent the largest available source of Gini coefficients. When no Gini coefficient was available for 2012, we used the most recent figure over the previous 10 years, with data available for 105 countries. Countries without Gini data in the last 10 years were excluded from the analysis (*n* = 26).

### National Income

LMIC were defined using World Bank country classifications as those with a Gross National Income (GNI) per capita of less than $12,736 in 2014, (*n* = 131) [27]. Gross domestic product (GDP) per capita in current $US obtained from the World Bank (data.data.worldbank.org) was used as our measure of national wealth [28]. To account for the delayed influence of national wealth on health outcomes, we calculated mean GDP over the previous 10 years from 2002 to 2012. All GDP data were log transformed due to non-normality.

### Statistical analysis

We undertook multi variable linear regression analyses to estimate the association of national wealth (log mean GDP over previous 10 years) and income inequality (Gini coefficient) with log mortality, separately by sex for each age-group, within LMIC in 2012. Models for Gini were adjusted for GDP and for the method used to derive the Gini estimate (income or consumption/expenditure, gross or net disposable income, and individual or household per capita income) [22]. To assess differences between the effect of country wealth and income inequality on mortality, we compared regression coefficients by age group. All analyses were performed in Stata 14 (StataCorp, College Station TX).

## Results

Mortality data were available for 188 countries, of which 131 were classified as LMIC. Of these, 103 (79%) also had data on both Gini coefficient and GDP and were included in the analysis.

**Fig. 1:**
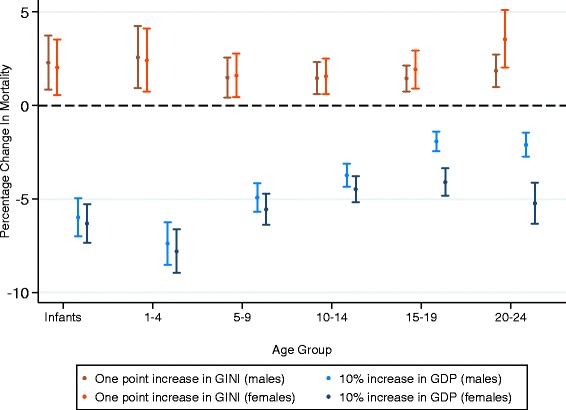
Percentage change in all-cause mortality from 10% increase in GDP or one unit increase in Gini (males and females)

**Fig. 2:**
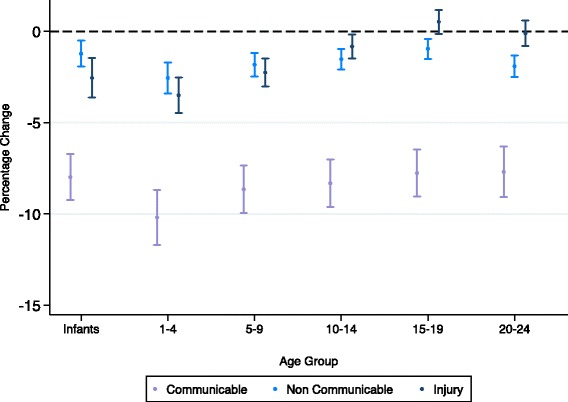
Percentage change in cause specific mortality from 10% increase in GDP (males)

**Fig. 3:**
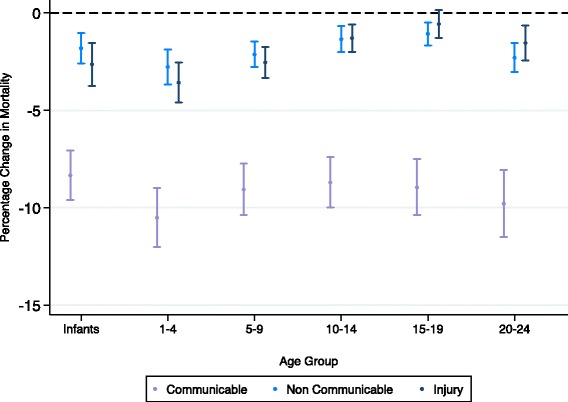
Percentage change in cause specific mortality from 10% increase in GDP (females)

GDP was negatively associated with all-cause, communicable, NCD and injury mortality for males and females (Figs. 1, 2 and 3). The strongest association was seen for communicable disease in 1–4 year olds, where a 10% increase in GDP was associated with a 10.2% (95% CI 11.7–8.7) and 10.5% (12.0–9.0) decrease in mortality rate for males and females respectively (see Tables S1 and S2 in Additional file 1: Appendix A). After infancy, as age increased the strength of these associations decreased in both males and females, reaching a nadir amongst 15–19 year olds and then increasing in 20–24 year olds. Trends of the strength of this association were similar for males and females up to mid adolescence (10–14), but then diverged, with males 15–24 benefiting much less than females from an increase in GDP. Coefficients for associations of mortality and GDP varied significantly by age for all-cause (*p* < 0.01 in both sexes), NCD (*p* < 0.01 in both sexes) and injury mortality ((*p* < 0.01 in both sexes) but not for communicable disease mortality.

**Fig. 4:**
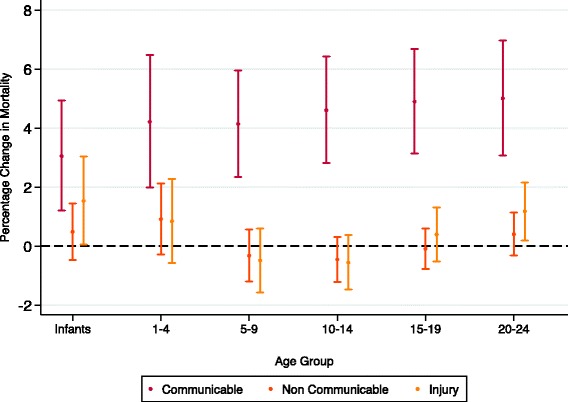
Percentage change in cause specific mortality from one unit increase in Gini (males)

**Fig. 5:**
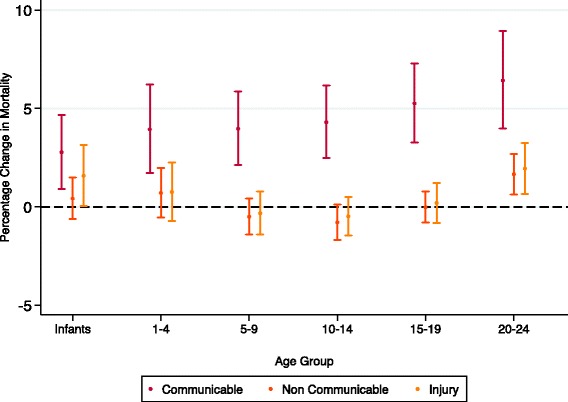
Percentage change in cause specific mortality from one unit increase in Gini (females)

Gini estimates ranged from 23.7 to 73.5 (see Additional file 1 for full table). Gini was positively associated with increased all-cause and communicable disease mortality for males and females across all age groups, after adjusting for mean GDP (Figs. 1, 4 and 5). The association was strongest amongst 20–24 year old females for communicable disease, where a one unit increase in Gini (increased inequality) was associated with a 6.4% (4–8.9) increase in communicable mortality rate (see Tables S3 and S4 in Additional file 1: Appendix B). Gini was not significantly associated with injury or NCD mortality, except amongst infants and 20–24 year olds for injury and 20–24 year old females for NCD mortality. After mid childhood (1–9), the strength of the association between Gini and all-cause and communicable mortality increased. These coefficients did not vary significantly by age group in either sex (see Additional file 1: Appendix C).

## Discussion

This is the first systematic study of income inequality, national wealth, and grouped cause child and adolescent mortality in LMIC. We found that both national wealth and national income inequality were strongly associated with mortality in children and young people, although in dramatically different ways. GDP was strongly associated with lower all-cause, communicable disease and NCD mortality for all age groups 0 to 24 years. The largest associations were for communicable diseases, with each additional 10% increase in GDP linked with a 7–10% fall in mortality across ages in both sexes. Changes in NCD mortality with GDP were of a much smaller order, and GDP was only associated with injury mortality in those under 14 years and females 20–24 years.

In contrast, national income inequality was strongly associated with higher all-cause and communicable disease mortality after adjustment for GDP, although not consistently associated with NCD or injury mortality. Income inequality appeared to play the greatest role in communicable disease mortality, with each point increase in Gini associated with a 3–6% increase mortality.

The pattern of associations for GDP and Gini across ages also differed markedly. GDP appeared to play a much greater role in mortality amongst infants and children than amongst young people. Each additional 10% increase in GDP was associated with a 6% reduction in all-cause mortality amongst infants but only 2% amongst young men. In contrast, effects of inequality were as high amongst young people as younger children for all cause mortality, but increased with age for maternal and communicable disease mortality throughout childhood.

If the relationship between national wealth and national income inequality on mortality is causative, the associations found here suggest the effect size is large. The median increase in GDP in the 10 years from 2002 to 2012 amongst LMIC was 160%. Our data suggest this level of economic growth would have been associated with a reduction in all-cause mortality of between 30 and 125%; communicable disease mortality of 123–168%; NCD mortality of between 15 and 44% and injury mortality of between 13 and 57%.

The median Gini change in our sample over the decade preceding 2012 was −2.1. Our data suggest this would be associated with a reduction in male and female all-cause mortality of 3–7%, communicable disease mortality of 5–13%, and for 20–24 year olds, a 2–4% reduction in injury mortality, *in addition* to the benefits attributed to increased GDP. Importantly, levels of inequality *increased* in 30 countries in our dataset during this time period. In these countries, the benefits to mortality of increased GDP may have been limited by the negative impact of increasing inequality, particularly amongst young men.

### Comparison with the literature

The only previous similar study was by Dorling et al. [4], who analysed variations in the association between national GDP, Gini coefficient and all-cause mortality by age in 126 countries in 2002. Similar to our findings, they reported that as age increased to late adolescence and early adulthood, the strength of the association between national wealth and mortality weakened and that between income inequality and mortality strengthened. Other studies have reported associations between wealth and inequality and mortality in infants [29] or in adolescents [30], however no previous studies have analysed mortality by high-level cause systematically across age-groups in LMIC (as here) or across all countries.

### Strengths and limitations

We were able to include data from 79% of all low and middle-income economies covering 97% of the global population of these countries. We used mean GDP over the previous 10 years as our measure for national wealth to account for rapid fluctuations in country GDP and the delayed effect of economic development on population health. 80% of the Gini coefficients we used were calculated within 5 years of 2012, the year we used for our mortality data. We studied high-level cause mortality rates, and undertook analyses separately by sex.

There were a number of limitations to our study. The use of Gini coefficients as a measure of inequality has been criticized as it is most sensitive to inequalities within the middle of the income spectrum, and does not capture different types of inequality within countries [26]. Where robust mortality data are not available, IHME include country GDP to model mortality estimates, which we included as a predictor for mortality. However, this would not have affected the associations between Gini coefficient and mortality reported here. Using contemporaneous Gini coefficients and mortality may not have captured any delayed effects of inequality on health, which some have suggested in previous studies [31]. This ecological analysis describes associations at the population level, which may not be replicated at the individual level. Further, our analyses are cross-sectional, and so we cannot comment on causality to explain the associations described here, for which longitudinal studies are needed.

### Meaning and mechanisms

Our findings relating to GDP confirm the relationship of economic development to the epidemiological transition [32] in this subset of LMIC. They suggest, however, that economic development benefits infants and young children to a much greater degree than adolescents and young adults. Further, we showed a sex difference emerging in adolescence and young adulthood, with the mortality benefits of economic development for young men being approximately half that of young women. This likely reflects the greater contribution of NCD and injury causes to mortality in young people, particularly young men, each of which are less influenced by wealth than communicable disease mortality. This is consistent with our previous report that global mortality declines amongst 15–24 year olds were notably lower than those seen in children under 10 years over the past 4 decades [33].

In contrast to wealth, we found national income inequality to be an important predictor of mortality across all age groups studied here, with this association strengthening with age. Multiple causal mechanisms have been proposed to explain the relationship between inequality and poor health, independent of the influence of absolute deprivation, and include individual, social and structural factors [34]. The “psychosocial” interpretation states that poor individual health stems from the perception of others both above and below oneself in the social and income hierarchy. Large differences within this hierarchy create “status anxiety”, leading to chronic stress and worse health outcomes [1]. Greater inequality is also thought to erode “social capital” within communities, known to be protective of poor health [35, 36], in part through reducing levels of trust and group membership [37] and increasing hostility, violence, racism, sexism and other forms of discrimination [38, 39]. This weakens social affiliations and further exacerbates the impact of low social status on health, and increases mortality [38]. There is also evidence that income inequality and may influence other structural determinants of health through exacerbating existing health inequalities [40] and weakening a society’s willingness to invest in improvements that promote wellbeing, such as welfare programmes, access to healthcare services, and education [41–43].

Considering these causal mechanisms, income inequality may be particularly harmful for adolescents and young adults. During this stage of the life course social factors at family, community and national level are particularly strong determinants for health [30], and the importance of how we are viewed by our peers is heightened [44]. Studies of income inequality which include adolescent outcomes are rare but appear to confirm this, with income inequality being positively associated with teenage pregnancy rate [6], risk of smoking cigarettes [45], HIV prevalence, bullying [30], lower levels of physical activity, higher body mass index, more psychological and physical symptoms [40], and lower life satisfaction [46]. Further, as female adolescents appear to be particularly vulnerable to the effects of income inequality [46], this may worsen maternal health and increase perinatal risk, particularly in low-income settings with high adolescent fertility rates [47].

## Conclusion

Our findings suggest that national wealth benefits younger children more than adolescents, whereas inequality is harmful for all age groups, and increases in importance with age. Improving health in adolescence in low and middle-income countries is increasingly recognized as a global health priority, [48] and our analysis suggests policies to reduce income inequality in these settings may be of particular benefit to this age group.

## Additional files

Additional file 1: Appendices A, B, C and D. 1) Regression coefficients for cause specific male mortality (log) and mean GDP (log) by age group and percentage change in mortality rate from 10% increase in mean GDP. 2) Regression coefficients for male cause specific mortality (log) and Gini coefficient adjusted for mean GDP by age group. Also showing percentage change in mortality rate from one unit increase in Gini coefficient (increased inequality). 3) How regression coefficients for Gini and GDP models predicting mortality amongst 20–24 year olds differed compared with younger age groups. 4) Mean GDP and Gini coefficients for low and middle-income countries in 2012. (DOCX 43 kb)

## Abbreviations

GBD: Global Burden of Disease
GDP: Gross Domestic Product
GNI: Gross National Income
HIV: Human Immunodeficiency Virus
IHME: Institute of Health Metrics and Evaluation
LMIC: Low and Middle Income Country
NCD: Non-communicable disease
OECD: Organization for Economic Cooperation and Development
UNDP: United Nations Development Programme
