# Supplementary material for: The impact of income inequality and national wealth on child and adolescent mortality in low and middle-income countries
Source: BMC Public Health. 2017 May 11;17:429. doi: 10.1186/s12889-017-4310-z (PMC5425964; doi:10.1186/s12889-017-4310-z)
Supplement: Supplementary file 1 — Appendices A, B, C and D. 1) Regression coefficients for cause specific male mortality (log) and mean GDP (log) by age group and percentage change in mortality rate from 10% increase in mean GDP. 2) Regression coefficients for male cause specific mortality (log) and Gini coefficient adjusted for mean GDP by age group. Also showing percentage change in mortality rate from one unit increase in Gini coefficient (increased inequality). 3) How regression coefficients for Gini and GDP models predicting mortality amongst 20–24 year olds differed compared with younger age groups. 4) Mean GDP and Gini coefficients for low and middle-income countries in 2012. (DOCX 43 kb) [file 12889_2017_4310_MOESM1_ESM.docx]

**Appendix A**

Table 1. Regression coefficients for cause specific male mortality (log) and mean GDP (log) by age group and percentage change in mortality rate from 10% increase in mean GDP.

| Males | Regression Coefficient and 95% CI | | | | Percentage Change in Mortality Rate for 10% increase in mean GPD | | |
| --- | --- | --- | --- | --- | --- | --- | --- |
|  | r | p | upper | lower | mean | upper | lower |
| All Cause |  |  |  |  |  |  |  |
| Infants | -0.65 | 0.00 | -0.76 | -0.53 | -5.98 | -6.99 | -4.97 |
| Ages 1-4 | -0.80 | 0.00 | -0.93 | -0.68 | -7.38 | -8.51 | -6.24 |
| Ages 5-9 | -0.53 | 0.00 | -0.61 | -0.44 | -4.92 | -5.69 | -4.14 |
| Ages 10-14 | -0.40 | 0.00 | -0.47 | -0.33 | -3.74 | -4.36 | -3.11 |
| Ages 15-19 | -0.20 | 0.00 | -0.26 | -0.15 | -1.92 | -2.44 | -1.40 |
| Ages 20-24 | -0.22 | 0.00 | -0.29 | -0.15 | -2.11 | -2.74 | -1.46 |
| Communicable |  |  |  |  |  |  |  |
| Infants | -0.87 | 0.00 | -1.02 | -0.73 | -7.99 | -9.25 | -6.72 |
| Ages 1-4 | -1.13 | 0.00 | -1.31 | -0.95 | -10.21 | -11.70 | -8.68 |
| Ages 5-9 | -0.95 | 0.00 | -1.10 | -0.80 | -8.66 | -9.96 | -7.35 |
| Ages 10-14 | -0.91 | 0.00 | -1.06 | -0.76 | -8.33 | -9.62 | -7.02 |
| Ages 15-19 | -0.85 | 0.00 | -1.00 | -0.70 | -7.77 | -9.06 | -6.47 |
| Ages 20-24 | -0.84 | 0.00 | -1.00 | -0.68 | -7.70 | -9.07 | -6.31 |
| Non - Communicable |  |  |  |  |  |  |  |
| Infants | -0.13 | 0.00 | -0.21 | -0.05 | -1.23 | -1.94 | -0.52 |
| Ages 1-4 | -0.27 | 0.00 | -0.36 | -0.18 | -2.57 | -3.40 | -1.72 |
| Ages 5-9 | -0.19 | 0.00 | -0.26 | -0.12 | -1.83 | -2.47 | -1.18 |
| Ages 10-14 | -0.16 | 0.00 | -0.22 | -0.10 | -1.53 | -2.09 | -0.96 |
| Ages 15-19 | -0.10 | 0.00 | -0.16 | -0.04 | -0.96 | -1.51 | -0.42 |
| Ages 20-24 | -0.20 | 0.00 | -0.27 | -0.14 | -1.91 | -2.50 | -1.33 |
| Injury |  |  |  |  |  |  |  |
| Infants | -0.27 | 0.00 | -0.39 | -0.16 | -2.56 | -3.64 | -1.47 |
| Ages 1-4 | -0.38 | 0.00 | -0.48 | -0.27 | -3.51 | -4.49 | -2.53 |
| Ages 5-9 | -0.24 | 0.00 | -0.32 | -0.16 | -2.26 | -3.02 | -1.50 |
| Ages 10-14 | -0.09 | 0.01 | -0.16 | -0.02 | -0.84 | -1.48 | -0.19 |
| Ages 15-19 | 0.05 | 0.12 | -0.01 | 0.12 | 0.51 | -0.14 | 1.16 |
| Ages 20-24 | -0.01 | 0.75 | -0.09 | 0.06 | -0.11 | -0.81 | 0.59 |

Table 2. Regression coefficients for cause specific female mortality (log) and mean GDP (log) by age group and percentage change in mortality rate from 10% increase in mean GDP.

| Females | Regression Coefficient and 95% CI | | | | Percentage Change in Mortality Rate for 10% increase in mean GDP | | |
| --- | --- | --- | --- | --- | --- | --- | --- |
|  | r | p | upper | lower | mean | upper | lower |
| All Cause |  |  |  |  |  |  |  |
| Infants | -0.68 | <0.00 | -0.80 | -0.57 | -6.32 | -7.33 | -5.29 |
| Ages 1-4 | -0.85 | <0.00 | -0.98 | -0.72 | -7.80 | -8.96 | -6.63 |
| Ages 5-9 | -0.60 | <0.00 | -0.69 | -0.51 | -5.56 | -6.39 | -4.72 |
| Ages 10-14 | -0.48 | <0.00 | -0.56 | -0.40 | -4.47 | -5.17 | -3.77 |
| Ages 15-19 | -0.44 | <0.00 | -0.52 | -0.36 | -4.10 | -4.82 | -3.37 |
| Ages 20-24 | -0.56 | <0.00 | -0.68 | -0.44 | -5.24 | -6.32 | -4.14 |
| Communicable |  |  |  |  |  |  |  |
| Infants | -0.91 | <0.00 | -1.06 | -0.77 | -8.34 | -9.59 | -7.07 |
| Ages 1-4 | -1.17 | <0.00 | -1.34 | -0.99 | -10.52 | -12.02 | -9.00 |
| Ages 5-9 | -1.00 | <0.00 | -1.15 | -0.84 | -9.06 | -10.38 | -7.73 |
| Ages 10-14 | -0.96 | <0.00 | -1.10 | -0.81 | -8.70 | -9.99 | -7.40 |
| Ages 15-19 | -0.98 | <0.00 | -1.15 | -0.82 | -8.96 | -10.38 | -7.51 |
| Ages 20-24 | -1.08 | <0.00 | -1.28 | -0.88 | -9.80 | -11.50 | -8.07 |
| Non - Communicable |  |  |  |  |  |  |  |
| Infants | -0.19 | <0.00 | -0.28 | -0.11 | -1.81 | -2.60 | -1.02 |
| Ages 1-4 | -0.29 | <0.00 | -0.39 | -0.20 | -2.77 | -3.67 | -1.86 |
| Ages 5-9 | -0.23 | <0.00 | -0.30 | -0.16 | -2.13 | -2.78 | -1.47 |
| Ages 10-14 | -0.14 | <0.00 | -0.21 | -0.07 | -1.35 | -2.02 | -0.68 |
| Ages 15-19 | -0.11 | <0.00 | -0.18 | -0.05 | -1.08 | -1.66 | -0.49 |
| Ages 20-24 | -0.24 | <0.00 | -0.32 | -0.16 | -2.29 | -3.03 | -1.55 |
| Injury |  |  |  |  |  |  |  |
| Infants | -0.28 | <0.00 | -0.40 | -0.16 | -2.64 | -3.74 | -1.53 |
| Ages 1-4 | -0.38 | <0.00 | -0.49 | -0.27 | -3.58 | -4.60 | -2.54 |
| Ages 5-9 | -0.27 | <0.00 | -0.36 | -0.18 | -2.54 | -3.33 | -1.74 |
| Ages 10-14 | -0.14 | <0.00 | -0.21 | -0.06 | -1.29 | -1.99 | -0.59 |
| Ages 15-19 | -0.06 | 0.12 | -0.14 | 0.02 | -0.57 | -1.29 | 0.15 |
| Ages 20-24 | -0.16 | <0.00 | -0.26 | -0.07 | -1.54 | -2.45 | -0.63 |

**Appendix B**

Table 3. Regression coefficients for male cause specific mortality (log) and Gini coefficient adjusted for mean GDP by age group. Also showing percentage change in mortality rate from one unit *increase* in Gini coefficient (increased inequality).

| Males | Regression Coefficient and 95% CI | | | | Percentage Change in Mortality Rate for 1 unit increase in Gini | | |
| --- | --- | --- | --- | --- | --- | --- | --- |
|  | r | p | upper | lower | mean | upper | lower |
| All Cause |  |  |  |  |  |  |  |
| Infants | 0.023 | <0.01 | 0.008 | 0.037 | 2.28 | 0.84 | 3.74 |
| Ages 1-4 | 0.025 | <0.01 | 0.009 | 0.041 | 2.56 | 0.92 | 4.23 |
| Ages 5-9 | 0.015 | 0.01 | 0.004 | 0.025 | 1.48 | 0.41 | 2.56 |
| Ages 10-14 | 0.014 | <0.01 | 0.006 | 0.023 | 1.46 | 0.61 | 2.30 |
| Ages 15-19 | 0.014 | <0.01 | 0.007 | 0.021 | 1.44 | 0.75 | 2.13 |
| Ages 20-24 | 0.018 | <0.01 | 0.010 | 0.027 | 1.84 | 0.97 | 2.71 |
| Communicable |  |  |  |  |  |  |  |
| Infants | 0.030 | <0.01 | 0.012 | 0.048 | 3.05 | 1.20 | 4.93 |
| Ages 1-4 | 0.041 | <0.01 | 0.020 | 0.063 | 4.21 | 1.99 | 6.48 |
| Ages 5-9 | 0.041 | <0.01 | 0.023 | 0.058 | 4.14 | 2.34 | 5.96 |
| Ages 10-14 | 0.045 | <0.01 | 0.028 | 0.062 | 4.60 | 2.82 | 6.42 |
| Ages 15-19 | 0.048 | <0.01 | 0.031 | 0.065 | 4.89 | 3.15 | 6.67 |
| Ages 20-24 | 0.049 | <0.01 | 0.030 | 0.067 | 5.01 | 3.08 | 6.97 |
| Non - Communicable |  |  |  |  |  |  |  |
| Infants | 0.005 | 0.32 | -0.005 | 0.014 | 0.48 | -0.47 | 1.44 |
| Ages 1-4 | 0.009 | 0.14 | -0.003 | 0.021 | 0.91 | -0.29 | 2.12 |
| Ages 5-9 | -0.003 | 0.47 | -0.012 | 0.006 | -0.33 | -1.21 | 0.56 |
| Ages 10-14 | -0.005 | 0.24 | -0.012 | 0.003 | -0.45 | -1.21 | 0.31 |
| Ages 15-19 | -0.001 | 0.80 | -0.008 | 0.006 | -0.09 | -0.77 | 0.60 |
| Ages 20-24 | 0.004 | 0.28 | -0.003 | 0.011 | 0.40 | -0.33 | 1.13 |
| Injury |  |  |  |  |  |  |  |
| Infants | 0.015 | 0.04 | 0.000 | 0.030 | 1.53 | 0.05 | 3.04 |
| Ages 1-4 | 0.008 | 0.24 | -0.006 | 0.022 | 0.84 | -0.57 | 2.27 |
| Ages 5-9 | -0.005 | 0.37 | -0.016 | 0.006 | -0.49 | -1.57 | 0.60 |
| Ages 10-14 | -0.006 | 0.23 | -0.015 | 0.004 | -0.56 | -1.48 | 0.37 |
| Ages 15-19 | 0.004 | 0.40 | -0.005 | 0.013 | 0.39 | -0.53 | 1.31 |
| Ages 20-24 | 0.012 | 0.02 | 0.002 | 0.021 | 1.17 | 0.19 | 2.16 |

Table 4. Regression coefficients for female cause specific mortality (log) and Gini coefficient adjusted for mean GDP by age group. Also showing percentage change in mortality rate from one unit *increase* in Gini coefficient (increased inequality).

| Females | Regression Coefficient and 95% CI | | | | Percentage Change in Mortality Rate for 1 unit increase in Gini | | |
| --- | --- | --- | --- | --- | --- | --- | --- |
|  | r | p | upper | lower | mean | upper | lower |
| All Cause |  |  |  |  |  |  |  |
| Infants | 0.020 | 0.01 | 0.006 | 0.035 | 2.02 | 0.55 | 3.51 |
| Ages 1-4 | 0.024 | 0.01 | 0.007 | 0.040 | 2.41 | 0.74 | 4.10 |
| Ages 5-9 | 0.016 | 0.01 | 0.004 | 0.027 | 1.59 | 0.44 | 2.76 |
| Ages 10-14 | 0.015 | <0.01 | 0.006 | 0.025 | 1.55 | 0.60 | 2.51 |
| Ages 15-19 | 0.019 | <0.01 | 0.009 | 0.029 | 1.92 | 0.91 | 2.94 |
| Ages 20-24 | 0.035 | <0.01 | 0.020 | 0.050 | 3.54 | 2.01 | 5.08 |
| Communicable |  |  |  |  |  |  |  |
| Infants | 0.027 | <0.01 | 0.009 | 0.046 | 2.77 | 0.90 | 4.66 |
| Ages 1-4 | 0.039 | <0.01 | 0.017 | 0.060 | 3.94 | 1.72 | 6.21 |
| Ages 5-9 | 0.039 | <0.01 | 0.021 | 0.057 | 3.96 | 2.11 | 5.85 |
| Ages 10-14 | 0.042 | <0.01 | 0.024 | 0.060 | 4.30 | 2.47 | 6.17 |
| Ages 15-19 | 0.051 | <0.01 | 0.032 | 0.070 | 5.25 | 3.26 | 7.28 |
| Ages 20-24 | 0.062 | <0.01 | 0.039 | 0.086 | 6.42 | 3.97 | 8.93 |
| Non - Communicable |  |  |  |  |  |  |  |
| Infants | 0.004 | 0.44 | -0.006 | 0.015 | 0.42 | -0.63 | 1.48 |
| Ages 1-4 | 0.007 | 0.27 | -0.006 | 0.020 | 0.70 | -0.56 | 1.98 |
| Ages 5-9 | -0.005 | 0.28 | -0.014 | 0.004 | -0.51 | -1.42 | 0.42 |
| Ages 10-14 | -0.008 | 0.08 | -0.017 | 0.001 | -0.80 | -1.69 | 0.10 |
| Ages 15-19 | 0.000 | 0.95 | -0.008 | 0.008 | -0.02 | -0.81 | 0.77 |
| Ages 20-24 | 0.016 | <0.01 | 0.006 | 0.027 | 1.65 | 0.63 | 2.69 |
| Injury |  |  |  |  |  |  |  |
| Infants | 0.016 | 0.04 | 0.000 | 0.031 | 1.58 | 0.04 | 3.13 |
| Ages 1-4 | 0.007 | 0.32 | -0.007 | 0.022 | 0.75 | -0.72 | 2.25 |
| Ages 5-9 | -0.003 | 0.55 | -0.014 | 0.008 | -0.33 | -1.42 | 0.77 |
| Ages 10-14 | -0.005 | 0.32 | -0.015 | 0.005 | -0.49 | -1.46 | 0.49 |
| Ages 15-19 | 0.002 | 0.71 | -0.008 | 0.012 | 0.19 | -0.82 | 1.22 |
| Ages 20-24 | 0.019 | <0.01 | 0.007 | 0.032 | 1.94 | 0.65 | 3.24 |

**Appendix C**

Table 5. How regression coefficients for Gini and GDP models predicting mortality amongst 20-24 year olds differed compared with younger age groups.

|  | Gini | | | | GDP | | | |
| --- | --- | --- | --- | --- | --- | --- | --- | --- |
|  | Males | | Females | | Males | | Females | |
|  | F | p | F | p | F | p | F | p |
| All Cause | 0.35 | 0.88 | 0.69 | 0.63 | 27.26 | <0.01 | 7.99 | <0.01 |
| Communicable | 0.40 | 0.85 | 1.14 | 0.34 | 1.91 | 0.09 | 1.21 | 0.30 |
| Non Communicable | 2.09 | 0.07 | 2.12 | 0.06 | 2.91 | 0.01 | 2.86 | 0.01 |
| Injury | 2.61 | 0.02 | 2.16 | 0.06 | 14.02 | <0.01 | 5.85 | <0.01 |

**Appendix D**

Table 6. Mean GDP and Gini coefficients for low and middle-income countries in 2012

|  | Gini coefficient^a^ | Mean GDP  (per capita current US$)^b^ |
| --- | --- | --- |
| **Sub-Saharan Africa** |  |  |
| Angola | 55.0 | 2630 |
| Benin | 34.1 | 596 |
| Botswana^*^ |  | 5282 |
| Burkina Faso | 39.8 | 463 |
| Burundi | 33.3 | 162 |
| Cameroon | 39.0 | 1005 |
| Cape Verde | 73.5 | 2698 |
| Central African Republic | 56.2 | 381 |
| Chad | 42.1 | 676 |
| Comoros | 62.9 | 668 |
| Congo | 38.4 | 2141 |
| Cote d'Ivoire | 44.7 | 1047 |
| Democratic Republic of Congo | 44.4 | 252 |
| Eritrea^*^ |  | 324 |
| Ethiopia | 33.3 | 237 |
| Gabon | 41.1 | 7679 |
| Gambia | 46.9 | 485 |
| Ghana | 42.8 | 888 |
| Guinea | 31.7 | 383 |
| Guinea-Bissau | 38.3 | 452 |
| Kenya | 29.9 | 730 |
| Lesotho | 52.5 | 778 |
| Liberia | 38.1 | 225 |
| Madagascar | 40.9 | 353 |
| Malawi | 45.2 | 276 |
| Mali | 33.0 | 495 |
| Mauritania | 40.5 | 905 |
| Mauritius^*^ |  | 6226 |
| Mozambique | 45.6 | 365 |
| Namibia | 59.7 | 3797 |
| Niger | 29.8 | 287 |
| Nigeria | 46.8 | 1186 |
| Rwanda | 50.2 | 385 |
| Sao Tome and Principe | 39.0 | 949 |
| Senegal | 33.8 | 861 |
| Sierra Leone | 32.1 | 378 |
| Somalia^*^ |  |  |
| South Africa | 66.5 | 5568 |
| South Sudan^*^ |  | 1555 |
| Sudan | 34.4 | 948 |
| Swaziland | 51.5 | 2411 |
| Tanzania | 37.6 | 525 |
| Togo | 40.9 | 425 |
| Uganda | 43.5 | 395 |
| Zambia | 57.4 | 977 |
| Zimbabwe | 42.3 | 502 |
| **South Asia** |  |  |
| Afghanistan | 27.8 | 357 |
| Bangladesh | 31.7 | 572 |
| Bhutan | 38.4 | 1566 |
| India | 35.5 | 928 |
| Maldives | 37.0 | 5215 |
| Nepal | 36.9 | 411 |
| Pakistan | 30.0 | 859 |
| Sri Lanka | 36.1 | 1654 |
| **Middle East & North Africa** |  |  |
| Algeria^*^ |  | 3566 |
| Djibouti | 39.7 | 1095 |
| Egypt | 29.9 | 1764 |
| Iran | 37.8 | 3971 |
| Iraq | 36.0 | 3410 |
| Jordan | 38.4 | 3096 |
| Lebanon^*^ |  | 6633 |
| Libya^*^ |  | 8472 |
| Morocco | 40.7 | 2270 |
| Palestine | 35.5 | 1705 |
| Syria | 32.0 | 1567 |
| Tunisia | 38.5 | 3574 |
| Yemen | 37.4 | 997 |
| **Latin America & Caribbean** |  |  |
| Belize^*^ |  | 4110 |
| Bolivia | 47.3 | 1435 |
| Brazil | 52.8 | 6857 |
| Colombia | 55.0 | 4317 |
| Costa Rica | 51.3 | 5902 |
| Cuba^*^ |  | 4592 |
| Dominica^*^ |  | 5979 |
| Dominican Republic | 49.0 | 4083 |
| Ecuador | 47.2 | 3571 |
| El Salvador | 46.8 | 3136 |
| Grenada^*^ |  | 6786 |
| Guatemala | 55.6 | 2344 |
| Guyana | 35.0 | 2011 |
| Haiti^*^ |  | 544 |
| Honduras | 57.5 | 1671 |
| Jamaica | 45.5 | 4464 |
| Mexico | 50.5 | 8244 |
| Nicaragua | 47.7 | 1309 |
| Panama | 53.1 | 5955 |
| Paraguay | 54.6 | 2224 |
| Peru | 46.2 | 3536 |
| Saint Lucia^*^ |  | 6143 |
| St. Vincent and Grenadines^*^ |  | 5544 |
| Suriname^*^ |  | 5408 |
| **Europe & Central Asia** |  |  |
| Albania | 30.4 | 3224 |
| Armenia | 27.5 | 2310 |
| Azerbaijan | 31.2 | 3415 |
| Belarus | 23.7 | 4107 |
| Bosnia and Herzegovina | 35.6 | 3524 |
| Bulgaria | 36.2 | 5007 |
| Georgia | 41.0 | 1963 |
| Kazakhstan | 26.7 | 5854 |
| Kyrgyz Republic | 34.0 | 672 |
| Macedonia | 43.6 | 3671 |
| Moldova | 33.1 | 1156 |
| Montenegro | 28.6 | 5020 |
| Romania | 29.7 | 6224 |
| Serbia | 27.0 | 4579 |
| Tajikistan | 30.5 | 498 |
| Turkey | 40.0 | 7787 |
| Turkmenistan | 39.2 | 2826 |
| Ukraine | 25.6 | 2348 |
| Uzbekistan | 35.2 | 839 |
| **East Asia & Pacific** |  |  |
| Cambodia | 28.1 | 589 |
| China | 47.4 | 2775 |
| Fiji | 42.8 | 3532 |
| Indonesia | 41.0 | 1903 |
| Kiribati^*^ |  | 1226 |
| Laos | 36.7 | 718 |
| Malaysia | 46.8 | 6722 |
| Marshall Islands^*^ |  | 2788 |
| Micronesia^*^ |  | 2499 |
| Mongolia | 36.5 | 1626 |
| Myanmar^*^ |  |  |
| North Korea^*^ |  |  |
| Papua New Guinea^*^ |  | 1017 |
| Philippines | 39.2 | 1565 |
| Samoa^*^ |  | 2855 |
| Solomon Islands^*^ |  | 1053 |
| Thailand | 39.3 | 3419 |
| Timor-Leste | 31.9 | 632 |
| Tonga^*^ |  | 2882 |
| Vanuatu^*^ |  | 2263 |
| Vietnam | 35.6 | 930 |

^*^Country not included in analysis (gini and/or mean gdp data not available).

^a^Gini coefficient for 2012 or most recent available in previous 10 years (2002 – 2012)

^b^Mean GDP over 10 years (2002-2012)
